# The Combined Effects of Hourly Multi-Pollutant on the Risk of Ambulance Emergency Calls: A Seven-Year Time Series Study

**DOI:** 10.3390/toxics11110895

**Published:** 2023-10-31

**Authors:** Hanxu Shi, Qiang Zhou, Hongjuan Zhang, Shengzhi Sun, Junfeng Zhao, Yasha Wang, Jie Huang, Yinzi Jin, Zhijie Zheng, Rengyu Wu, Zhenyu Zhang

**Affiliations:** 1 Department of Global Health, School of Public Health, Peking University, Beijing 100191, China; handsomegaoaixu@163.com (H.S.); yzjin@bjmu.edu.cn (Y.J.); zhengzj@bjmu.edu.cn (Z.Z.); 2 Shenzhen Center for Prehospital Care, Shenzhen 518025, China; sumintao0627@163.com (Q.Z.); beautifulxuaigao@163.com (H.Z.); 3 School of Public Health, Capital Medical University, Beijing 100054, China; shengzhisun@ccmu.edu.cn; 4 School of Computer Science, Peking University, Beijing 100871, China; zhaojf@pku.edu.cn; 5 National Engineering Research Center of Software Engineering, Peking University, Beijing 100871, China; wangys@sei.pku.edu.cn; 6 School of Public Health and Emergency Management, Southern University of Science and Technology, Shenzhen 518055, China; jiehuang001@hotmail.com; 7 Institute for Global Health and Development, Peking University, Beijing 100871, China

**Keywords:** multipollutant, ambulance emergency calls (AECs), environmental epidemiology

## Abstract

Background: Ambulance emergency calls (AECs) are seen as a more suitable metric for syndromic surveillance due to their heightened sensitivity in reflecting the health impacts of air pollutants. Limited evidence has emphasized the combined effect of hourly air pollutants on AECs. This study aims to investigate the combined effects of multipollutants (i.e., PM_2.5_, PM_10_, Ozone, NO_2_, and SO_2_) on all-cause and cause-specific AECs by using the quantile g-computation method. Methods: We used ambulance emergency dispatch data, air pollutant data, and meteorological data from between 1 January 2013 and 31 December 2019 in Shenzhen, China, to estimate the associations of hourly multipollutants with AECs. We followed a two-stage analytic protocol, including the distributed lag nonlinear model, to examine the predominant lag for each air pollutant, as well as the quantile g-computation model to determine the associations of air pollutant mixtures with all-cause and cause-specific AECs. Results: A total of 3,022,164 patients were identified during the study period in Shenzhen. We found that each interquartile range increment in the concentrations of PM_2.5_, PM_10_, Ozone, NO_2_, and SO_2_ in 0–8 h, 0–8 h, 0–48 h, 0–28 h, and 0–24 h was associated with the highest risk of AECs. Each interquartile range increase in the mixture of air pollutants was significantly associated with a 1.67% (95% CI, 0.12–3.12%) increase in the risk of all-cause AECs, a 1.81% (95% CI, 0.25–3.39%) increase in the risk of vascular AECs, a 1.77% (95% CI, 0.44–3.11%) increase in reproductive AECs, and a 2.12% (95% CI, 0.56–3.71%) increase in AECs due to injuries. Conclusions: We found combined effects of pollutant mixtures associated with an increased risk of AECs across various causes. These findings highlight the importance of targeted policies and interventions to reduce air pollution, particularly for PM, Ozone, and NO_2_ emissions.

## 1. Introduction

Air pollution is the leading cause of enormous public health burdens due to substantial excess morbidity and mortality [1]. Based on the 2019 Global Burden of Disease (GBD), ambient air pollution is a major determinant of disease burden, particularly in relation to cardiovascular disease-related morbidity and death [2]. Previous studies have established a correlation between short-term exposure to air pollutants and the incidence of cardiovascular disease, respiratory disease (i.e., respiratory tuberculosis; pneumonia; pulmonary embolism), malignant neoplasm of the trachea, bronchus and lung disease, and even death [3]. For example, a time-stratified study demonstrated that exposure to different particular metrics was associated with nonfatal myocardial infarction in Germany, from 2005 to 2015 [4]. Another 12-year study conducted in metropolitan France showed that long-term exposure to particular matter (PM) pollutants was associated with all-cause mortality [5]. However, evidence examining the associations of multipollutants with disease onset is still limited and inconclusive. Moreover, although the association between disease onset and air pollutants was determined by conducting multipollutant models in such limited previous studies, most multipollutant models were used to examine the robustness of the pollutant of interest by including other pollutants as covariates in the regression model [6,7]. Notably, previous studies have not yet assessed the predominant lag period for each air pollutant; therefore, an inaccurate lag period would bias the results in the multipollutant model. 

Ambulance emergency calls (AECs) are a more suitable metric for syndromic surveillance due to their heightened sensitivity in reflecting the health impacts of air pollutants [8,9]. They have also been regarded as a promising alternative to other types of administrative health data for determining the impacts of air pollution because the exact location (in latitude and longitude) and time (in minutes and seconds) of the dispatch call are recorded in the AECs dataset [10]. Although some previous studies have examined the association between air pollutants and AECs, these findings were inconsistent because of inaccurate disease grouping criteria [8,11]. For example, Chen et al. found that PM pollutants and SO_2_ were positively associated with the risk of AECs in Shenzhen city, China [8], but not in Ichiki et al.’s study in Japan [11] and Straney et al.’s study in Perth, Australia, [12] due to the use of different lag periods and performing varied study designs. In addition, different patterns of AECs can be attributed to different seasons and daytime/nighttime periods. Identifying these potential patterns can be useful in helping people avoid exposure, but more evidence is needed to clarify whether these patterns will trigger air pollutant-related AECs.

Given the scarce evidence determining the association between muti-pollutant and well-grouped AECs with an accurate lag period, as well as the lack of the classification of potential patterns (i.e., seasons and daytime/nighttime) to trigger pollutant-related AECs, it is pivotal to concentrate on these research gaps in this study. Thus, we hypothesized that short-term air pollutants could jointly increase the number of cause-specific AECs. Therefore, we conducted a distributed lag nonlinear model (DLNM) to examine the predominant lag for each air pollutant (i.e., PM_2.5_, PM_10_, Ozone, NO_2_, and SO_2_); then, we adopted the quantile g-computation model to estimate the combined effects of multipollutant on all-cause and cause-specific AECs.

## 2. Materials and Methods

### 2.1. Study Participants

The Shenzhen Center for Prehospital Care established a system consisting of 120 Emergency Medical Services (EMS) in 1994, comprising 73 emergency health institutions and 103 emergency stations. We used ambulance emergency dispatch data from 1 January 2013 to 31 December 2019 that were obtained from the Shenzhen Ambulance Emergency Center. We included patients aged 18 and older who utilized ambulance services for hospital transportation between 2012 and 2019 and had documented transfer time records. We excluded those with incomplete dispatch or delivery times, ambiguous prehospital diagnoses, or missing diagnostic data. Additionally, repeated calls from the same individuals were excluded.

### 2.2. Outcome Ascertainment

Our primary outcome was all-cause AECs, which we further classified into four cause-specific categories, including AECs due to vascular, respiratory, reproductive diseases, and injuries. For instance, vascular AECs include conditions such as stroke, hypertension, acute ischemic stroke, myocardial infarction, ischemic heart disease, acute coronary syndrome, and unstable angina. Calls pertaining to lung diseases, bronchitis, respiratory difficulties, and asthma were grouped as respiratory-related AECs. Those relating to pregnancy, abortion, or other reproductive disorders were classified as reproduction-related AECs. Calls associated with conditions like fracture, edema, dislocation, and traffic injuries were categorized as injury-related AECs. Furthermore, we devised a distinct category for AECs stemming from poisoning, mental health disorders, endocrine disorders, and digestive disorders. 

### 2.3. Air Pollutant Exposure Ascertainment and Covariates

In this study, we obtained the hourly average city-level air pollutant concentration as the exposure for patients from the National Environmental Monitor Station during the study period. There are 23 city-level environmental monitoring stations in Shenzhen, which are located in Futian, Nanshan, Luohu, and other districts (Figure S1). We conducted a well-established distributed lag nonlinear model (DLNM) for nonlinear concentration–response and nonlinear lag–response functions to determine the predominant lag period, and we calculated the mean concentration of the predominant lag for each air pollutant as our exposure to be used in the quantile g-computation model [13,14,15]. We defined each interquartile range (IQR) increment as the difference between the 25th and 75th centiles for each air pollutant. The primary model adjusted covariates as follows: an indicator day-of-week variable to account for short-term weekly variation; natural spline functions with 3 degrees of freedom (df) for temperature and relative humidity [13]. 

We collected the relevant demographic information such as age, sex, and admission date for each diagnosis. In our study, the cool season spans from 1 November to 31 March, while the warm season is from 1 April to 31 October. We segmented the time of day into daytime (07:00–19:00) and nighttime (20:00–6:00). New Year’s Day, Chinese New Year’s Day, Dragon Boat Festival, Tomb-Sweeping Day, Labour Day, and National Day were regarded as Chinese public holidays. We used an indicator variable for the day of the week to account for the short-term weekly variations. We generated a ‘day of year’ variable (0–365 days) to consider underlying temporal trends. In addition, we gathered meteorological data, including three-hour temperature and three-hour relative humidity, from 2013 to 2019 in Shenzhen from the World Meteorological Database.

### 2.4. Statistical Analysis

We followed a two-stage analytic protocol to estimate the associations between combined air pollutants (i.e., PM_2.5_, PM_10_, Ozone, NO_2_, and SO_2_) and all-cause AECs as well as cause-specific AECs (i.e., vascular diseases, respiratory diseases, reproduction illnesses, and injuries), which included the distributed lag nonlinear model (DLNM) and the quantile g-computation model. 

In the first stage, we determined the association of each air pollutant with AECs by using conditional logistic regression models to estimate odds ratios (ORs) with 95% confidence intervals (CIs) and examined the predominant lag for each air pollutant. We modeled the concentration–response function using a natural cubic B spline with 2 knots to account for potential nonlinear relationships and modeled the lag–response function using a linear function with 2 knots placed on the log scale of lags up to 48 h [10,16,17]. 

In the second stage, we assessed the associations of air pollutant mixtures with all-cause and cause-specific AECs by conducting quantile g-computation models. Quantile g-computation has been regarded as a simple and efficient statistical method for evaluating the combined effect of air pollutant mixtures when all pollutants simultaneously increase each quantile, which allows the combined air pollutants to present positive, negative, and null effects simultaneously [18,19]. In this study, we implemented this model by categorizing PM_2.5_, PM_10_, Ozone, NO_2_, and SO_2_ into quantiles, coded as 0, 1, 2, 3, and 4, respectively. The quantile g-computation model equation is as follows (confounders Z could also be included):

$$Y_{i}=\beta_{0}+\psi \sum_{j=1}^{p}w_{j}X_{j}^{q}+\varepsilon_{0}=\beta_{0}+\sum_{j=1}^{p}\beta_{j}X_{j}^{q}+\varepsilon_{0}$$

where $\sum_{j=1}^{p}\beta_{j}=\psi$ and each air pollutant is given a negative or positive weight. The quantile g-computation estimator of the exposure–response relationship is the sum of the regression coefficients across the included exposures [20]. Afterward, the excessive relative risk (ERR) with 95% CIs expressed the associations (excessive relative risk indicated that the risk of all-cause AECs resulted from each interquartile range increment of the combined effect of the mixture of air pollutants). The formula for ERR is as follows:

$$ERR=\left[\exp\left(\beta \right)-1\right]\times 100\%$$

Apart from this, we also conducted a subgroup analysis according to sex (male, female), age group (<20 years old, 20~64 years old, ≥65 years old), and season to determine the associations in a variety of subgroups.

In the sensitivity analysis, we further generated the nonlinear and non-homogeneous quantile g-computation model to check the stability of the results. Moreover, we extracted representative subtypes of vascular diseases (i.e., myocardial infarction, acute coronary syndromes, and stroke) and respiratory diseases (pneumonia, asthma, and upper respiratory infectious), as well as those associated significantly with air pollutants, to explore the potential differences among the main diseases. All analyses were performed in R version 4.2.1, and the statistical significance was set at a two-tailed *p*-value < 0.05.

## 3. Results

There were 3,022,164 AECs between 2013 and 2019 in Shenzhen. Within these AECs, 636,288, 137,960, 208,408, and 1,324,656 calls corresponded to vascular diseases, respiratory diseases, reproductive illnesses, and injuries, respectively. For all-cause AECs, 64.3% were from males, 81.5% were aged between 20 and 64 years, and 51.4% of calls occurred during daytime hours. Higher percentages of vascular and respiratory disease-related AECs (25.7% and 36.1%) were observed among older populations. A total of 77.9% of females made AECs due to reproductive illnesses, and 69.5% of males made AECs due to injuries. A total of 61.9% of reproductive-related AECs occurred during the nighttime. As expected, the population who used ambulance services increased from 2013 to 2019 (2013: 11.8%; 2014: 12.7%; 2015: 13.2%; 2016: 14.2%; 2017: 15.4%; 2018: 15.7%; 2019: 17.0%). Apart from this, the mean (SD) concentrations of air pollutants (PM_2.5_: 30.1 ± 18.7 μg/m^3^; PM_10_: 49.3 ± 27.1 μg/m^3^; Ozone: 60.5 ± 24.6 μg/m^3^; NO_2_: 32.0 ± 12.4 μg/m^3^; SO_2_: 19.7 ± 10.0 μg/m^3^), temperature (23.4 ± 5.8 °C), and relative humidity (69.8 ± 14.9%) are provided in Table 1. Low to moderate correlations were observed among air pollutants and meteorological factors (Spearman correlation r < 0.80) (Figure S2).

The risk of AECs increased consistently with increasing concentrations of air pollutants (i.e., PM_2.5_, PM_10_, Ozone, NO_2_, and SO_2_), showing a nearly linear relationship with no apparent thresholds (*p*-value < 0.001; Figure S3). In general, steep slopes representing the risk of AECs were observed when exposed to the accumulated concentration of air pollutants. In the models with different lags, we found associations of exposure to PM_2.5_ and PM_10_, with a higher incidence of AECs occurring in the concurrent hour. Conversely, Ozone, NO_2_, and SO_2_ were associated with an increased risk of AECs during prolonged periods (Figure S4). We found that each IQR increment in the concentrations of PM_2.5_ (24.0 µg/m^3^), PM_10_ (34.0 µg/m^3^), Ozone (46.0 µg/m^3^), NO_2_ (18.0 µg/m^3^), and SO_2_ (3.0 µg/m^3^) in 0–8 h, 0–8 h, 0–48 h, 0–28 h, and 0–24 h was associated with the highest risk of AECs (1.6%, 95% CI, 0.9–2.4%; 1.9%, 1.2–2.6%; 2.1%, 1.3–2.8%; 1.4%, 95% CI, 0.8–2.0%; 0.5%, 0.2–0.7%). Thus, we decided to use 0 to 8 h for PM_2.5_ and PM_10_, 0–48 h for Ozone, 0–28 h for NO_2_, and 0–24 h for SO_2_ to obtain the risk estimates (Figures S3 and S4).

**Figure 1:**
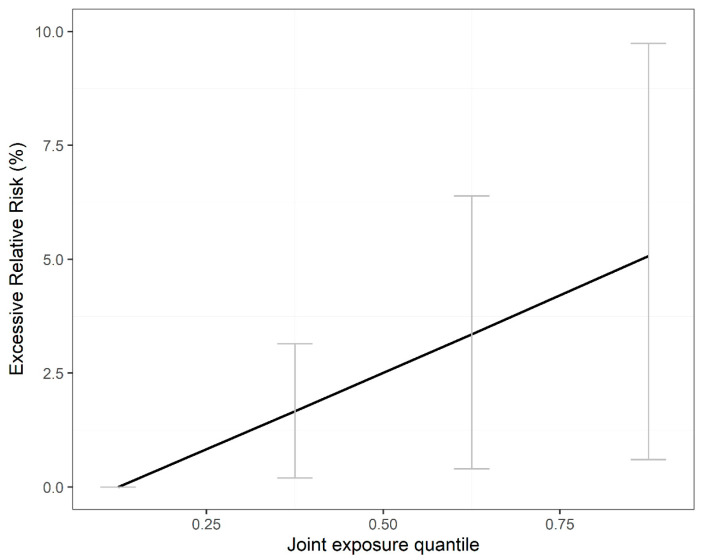
Exposure–response relationship between air pollutant mixtures and all-cause ambulance emergency calls in Shenzhen from 2013 to 2019. The line is the exposure–response curve, and the grey error bar is the confidence interval at each percentile. *p*-value < 0.001 for linear relationship.

**Figure 2:**
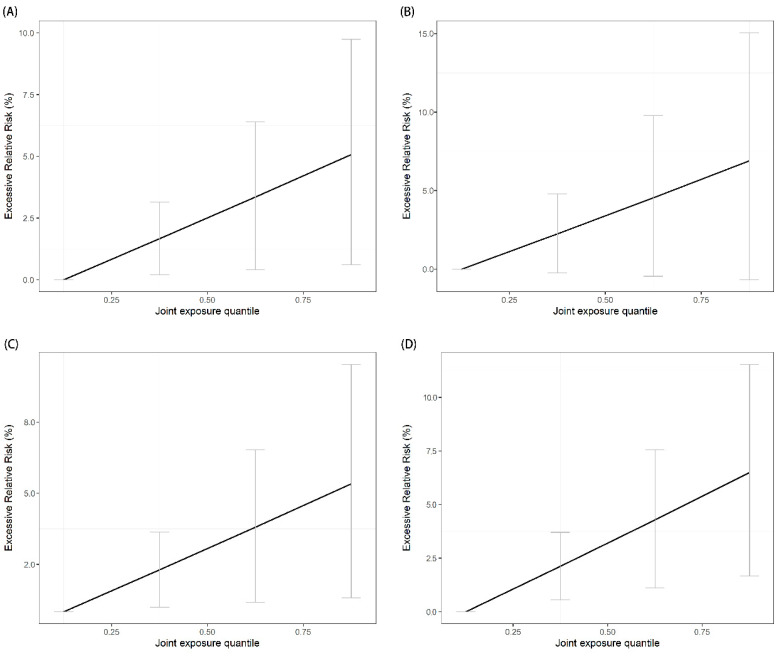
Exposure–response relationship between air pollutant mixtures and cause-specific ambulance emergency calls in Shenzhen from 2013 to 2019: (**A**) represents vascular disease (*p*-value < 0.001 for linear relationship); (**B**) represents respiratory disease (*p*-value < 0.001 for linear relationship); (**C**) represents reproductive disease (*p*-value < 0.001 for linear relationship); (**D**) represents injury (*p*-value < 0.001 for linear relationship). The line is the exposure–response curve, and the grey error bar is the confidence interval at each percentile.

We observed an approximately linear relationship between the combined exposure of air pollutants (i.e., PM_2.5_, PM_10_, Ozone, NO_2_, and SO_2_) and all-cause AECs or cause-specific AECs (Figure 1 and Figure 2). Each IQR increase in the mixture of PM_2.5_, PM_10_, Ozone, NO_2_, and SO_2_ was significantly associated with a 1.67% (95% CI, 0.12–3.12%) increase in the risk of all-cause AECs, a 1.81% (95% CI, 0.25–3.39%) increase in the risk of vascular-related AECs, a 2.25% (95% CI, −0.15–4.70%) increase in the risk of respiratory-related AECs, a 1.77% (95% CI, 0.44–3.11%) increase in reproduction-related AECs, and a 2.12% (95% CI, 0.56–3.71%) in AECs due to injuries (Table 2). 

The combined effect of the air pollutant mixture included a relatively predominant positive effect, with the sum of positive coefficients (β) equaling 0.0376, and a relatively slight negative effect, with the sum of negative coefficients (β) equaling −0.0204 (Figure S5). PM_10_ (45.5%), NO_2_ (23.4%), and Ozone (13.3%) exhibited positive weights with the same direction in the overall effect of air pollutants, whereas negative weights were observed among PM_2.5_ (52.6%) and SO_2_ (22.4%). 

We observed that the combined effect of PM_2.5_, PM_10_, Ozone, NO_2_, and SO_2_ was significant in some subgroups, including all-cause AECs in the age subgroup and cause-specific AECs in the time of the day subgroup (Table 3). The estimated ERRs of the combined exposure in the subgroup of older patients [1.89% (95% CI, 0.17–3.62%)] towards all-cause AECs, vascular-related [1.87% (95% CI, 0.00–3.82%)] AECs, and respiratory-related [2.23% (95% CI, −0.74–5.16%)] AECs were 1.17, 1.06, and 1.05 times higher than those in the younger group, respectively. Furthermore, the estimated ERRs for vascular-related [2.00% (95% CI, 0.42–2.56%)] and respiratory-related [2.53% (95% CI, 0.81–4.56%)] AECs in the daytime subgroup were higher than those AECs in the nighttime. 

We utilized nonlinear and nonhomogeneous quantile g-computation models (Figures S6 and S7) to validate the findings of the original nonlinear quantile g-computation models (Figure 1 and Figure 2). The results demonstrated an approximately linear relationship between combined exposure to air pollutants and AECs that was consistent with the original analysis (*p*-value < 0.001). Additionally, we conducted clearer and more specific analyses focusing on vascular patients utilizing ambulance emergency services for myocardial infarction (n = 2770), acute coronary syndromes (n = 30,892), and stroke (n = 92,302) (Figure S8), as well as respiratory patients experiencing AECs related to pneumonia (n = 31,159), asthma (n = 13,685), and upper respiratory infections (n = 33,305) (Figure S9). These additional analyses revealed that cause-specific AECs consistently increased with each interquartile range (IQR) of combined air pollutant exposure, aligning with the findings of the main analysis.

## 4. Discussion

In our large-scale study in Shenzhen, China, we found that exposure to PM_2.5_, PM_10_, Ozone, NO_2_, and SO_2_ was associated with an increased risk of the acute exacerbation of chronic conditions (AECs). The associations were significant in the older population and varied by time of day. These findings have important implications for developing an early warning system to mitigate pollutant-related AECs in the context of global climate change. 

Our study uniquely examines the combined effects of air pollutants on all-cause and cause-specific AECs using hourly air pollutant data, which provide valuable insights for medical rescue operations. This information is beneficial for emergency departments in optimizing ambulance arrangements and reducing response time delays. By understanding the specific effects of air pollutants on AECs at an hourly level, healthcare providers can enhance their preparedness and response strategies, ultimately improving patient outcomes. The findings from our study align with a robust body of previous research, consistently demonstrating positive associations between hourly air pollutants and the all-cause and cause-specific acute exacerbation of chronic conditions (AECs) [8,9,21,22,23,24]. For example, a time-stratified study by Chen et al. in Shenzhen, China, showed that PM_10_ and PM_2.5_ had immediate adverse effects on hourly AECs, persisting for 5 h; meanwhile, Ozone exhibited a longer lag pattern [8]. Similarly, a study in Luoyang City, China, found that PM_2.5_ and PM_10_ had acute health effects on all-cause morbidity at the current hours, while NO_2_ and SO_2_ were positively associated with all-cause morbidity at the lag period exceeding 18 h [9]. Another study by Liu et al. showed that exposure to PM_2.5_ significantly elevated the risk of all-cause AECs, as well as respiratory- and cardiovascular-related AECs in Chengdu, China, between 2013 and 2015 [21]. Moreover, Rao et al. conducted a case-crossover study by using British Columbia Emergency Health Service data from 2010 to 2015 in Canada, which showed a positive association between PM_2.5_ exposure and cardiovascular disease-related AECs, as well as a slight increase in respiratory disease-related AECs within 48 h [22]. 

Existing evidence primarily focuses on single- and two-pollutant models when analyzing the association [6,7,25,26,27,28]. However, it is crucial to examine the combined effects of hourly air pollutants on the risk of AECs, considering that humans are typically exposed to multiple pollutants simultaneously in the real world. In this study, we employed the quantile g-computation model to assess the combined effects of hourly air pollutant mixtures and identify the contribution of each pollutant. Previous studies used the same statistical approach [29,30], but they mainly focused on long-term air pollutants and cardiovascular morbidity and mortality. For example, Li et al. conducted a cohort study involving 31,162 individuals, demonstrating that exposure to a mixture of air pollutants increased the risk of 10-year atherosclerotic cardiovascular diseases, with PM pollutants accounting for 54% of the mixture effect, followed by Ozone at 42% [29]. Another study including 56,090 person-years found that a quartile increment in the mixture of Ozone, PM_2.5_, PM_1_, and NO_2_ was significantly associated with a 137% (95% CI: 130%, 144%) increase in cardiovascular risk in the Beijing–Tianjin–Hebei region during the period from 2017 to 2021 [30]. 

Our study revealed a positive association between exposure to hourly air pollutant mixtures and vascular-related AECs. This could be explained by air pollutants increasing the calcification of coronary arteries and the intima-media thickness of the common carotid artery, further resulting in the rupture of atheromatous plaques or the formation of thrombosis [31,32]. Given that air pollution has been regarded as an unavoidable environmental disturbance for vehicle traffic and might cause traffic accidents by worsening drivers’ physical and psychological conditions, another finding that exposure to a mix of air pollutants increased the risk of injury-related AECs in our study was consistent with this explanation [33]. 

In the subgroup analysis, we observed stronger associations of cause-specific AECs in the daytime, which could be explained by the fact that people often spend more time outdoors during the day and that they were estimated based on fixed-site monitors [13]. Moreover, there were no associations of air pollutants with AECs stratified by sex in our study. These results were in line with previous literature [34,35], indicating that all patients, regardless of sex, seem to be at higher risk of AECs after transient exposure to air pollution.

This study has some limitations. Firstly, we used the individual average hourly air pollution concentrations as a proxy for personal exposure. Although this approach may introduce some degree of exposure misclassification and potential underestimation of associations, it is a common limitation in most time-series studies [35,36]. Secondly, we relied on prehospital diagnoses based on para-medical records due to the unavailability of data on the final clinical diagnoses. However, we defined the subtypes of AECs using the Medical Priority Dispatch System (MPDS) [37], which helps mitigate significant biases in our results. Third, due to the unavailability of hourly pollutant data from individual monitors and the absence of patients’ residential addresses, we relied on aggregated pollutant data from multiple monitoring stations as provided by the National Environmental Monitor Station, rather than a single monitor. Nonetheless, this is unlikely to introduce significant measurement bias because Shenzhen, not being an industrial city, lacks major pollutant sources, and there are no significant differences in the annual concentrations of air pollutants across the various monitors.

This study also has several strengths. Firstly, the Shenzhen Ambulance Emergency Center provided high-quality data covering most hospitals in Shenzhen. The large sample size and individual-level data allowed for comprehensive statistical analyses, enhancing the validity of the study results. Secondly, the time-stratified, case-crossover study design with hourly data on air pollutants enables us to make more robust causal inferences by effectively adjusting for time-invariant confounding factors. Finally, we performed a quantile g-computation statistical strategy to examine the associations of pollutant mixtures with AECs, shedding light on the contribution of each pollutant to the risk of AECs and providing evidence for the urgent need to control specific pollutants.

Given the increasing prevalence of multiple air pollution-related AECs, the considerable cost of treatment, and the substantial adverse effects on individuals and society, local government response systems should typically provide air pollution-related information to the public, develop strategies to reduce air pollution-related risks, and allocate emergency ambulance resources equitably. Our findings benefit emergency departments in refining ambulance allocations and curtailing response time delays.

## 5. Conclusions

Our findings revealed distinct predominant lags for each pollutant. The combined effects of these pollutant mixtures were associated with an increased risk of both all-cause and cause-specific AECs. The risk increased linearly as pollutant concentrations increased, with no evident thresholds. Moreover, these associations were more pronounced in the older population and exhibited variations based on the time of day. Local governments must take the initiative to disseminate air pollution-related information to the public, develop strategies to minimize associated risks, and ensure an equitable distribution of emergency ambulance resources. Simultaneously, individual actions can also contribute significantly to diminishing the health impacts of air pollution. Future research might pivot towards examining individual behaviors and promoting personal air pollution monitoring.

## Figures and Tables

**Table 1:** Baseline characteristics in Shenzhen from 2013 to 2019.

	All	Vascular Disease	Respiratory Disease	Reproductive Illness	Injury	Others ^a^
**Cases**	3,022,164	636,288	137,960	208,408	1,324,656	714,852
**Age, years**						
Less than 20	185,152 (6.1)	35,412 (5.6)	19,836 (14.4)	8172 (3.9)	84,764 (6.4)	36,968 (5.2)
20 to 64	2,462,968 (81.5)	437,064 (68.7)	68,252 (49.5)	193,904 (93.0)	1,130,360 (85.3)	633,388 (88.6)
Equal or more than 65	373,884 (12.4)	163,796 (25.7)	49,852 (36.1)	6304 (3.1)	109,464 (8.3)	44,468 (6.2)
**Sex**						
Male	1,942,832 (64.3)	372,092 (58.5)	80,760 (58.5)	46,016 (22.1)	920,836 (69.5)	523,128 (73.2)
Female	1,079,256 (35.7)	264,188 (41.5)	57,196 (41.5)	162,380 (77.9)	403,780 (30.5)	191,712 (26.8)
**Cases by year**						
2013	355,816 (11.8)	70,340 (11.1)	15,172 (11.0)	26,784 (12.9)	168,940 (12.8)	74,580 (10.4)
2014	385,232 (12.7)	76,648 (12.0)	15,804 (11.5)	29,292 (14.1)	179,432 (13.5)	84,056 (11.8)
2015	399,756 (13.2)	83,152 (13.0)	16,760 (12.1)	26,704 (12.8)	185,484 (14.0)	87,656 (12.3)
2016	429,948 (14.2)	90,872 (14.3)	19,704 (14.3)	30,792 (14.7)	192,880 (14.6)	95,700 (13.4)
2017	465,060 (15.4)	96,648 (15.2)	20,748 (15.0)	31,232 (15.0)	204,800 (15.5)	111,632 (15.6)
2018	475,104 (15.7)	102,792 (16.2)	22,464 (16.3)	31,248 (15.0)	196,036 (14.7)	122,564 (17.1)
2019	511,248 (17.0)	115,836 (18.2)	27,308 (19.8)	32,356 (15.5)	197,084 (14.9)	138,664 (19.4)
**Time of day ^b^**						
Daytime	1,553,480 (51.4)	381,668 (60.0)	77,084 (55.9)	79,484 (38.1)	711,324 (53.7)	219,124 (30.7)
Nighttime	1,468,684 (48.6)	254,620 (40.0)	60,876 (44.1)	128,924 (61.9)	613,332 (46.3)	495,728 (69.3)
**Pollutants, μg/m** **^3^**						
PM_2.5_	30.1 ± 18.7	30.0 ± 18.6	29.4 ± 18.5	29.5 ± 18.4	29.7 ± 18.4	29.9 ± 18.5
PM_10_	49.3 ± 27.1	49.2 ± 27.1	48.5 ± 26.9	48.5 ± 26.7	48.9 ± 26.6	49.2 ± 27.0
Ozone	60.5 ± 24.6	60.6 ± 24.7	60.4 ± 24.9	60.4 ± 24.9	60.4 ± 24.8	60.6 ± 24.8
NO_2_	32.0 ± 12.4	31.9 ± 12.3	31.7 ± 12.4	31.6 ± 12.2	32.0 ± 12.4	31.9 ± 12.3
SO_2_	19.7 ± 10.0	19.7 ± 10.0	19.3 ± 9.9	19.3 ± 9.8	19.6 ± 9.9	19.6 ± 9.9
**Meteorology**						
Temperature, °C	23.4 ± 5.8	23.5 ± 5.8	23.6 ± 5.8	23.5 ± 5.8	23.9 ± 5.7	23.4 ± 5.8
Relative humidity, %	69.8 ± 14.9	69.4 ± 14.9	69.2 ± 14.7	71.0 ± 14.4	68.8 ± 14.9	70.1 ± 14.7

Abbreviations: PM_2.5_, particulate matter 2.5 µm in diameter; PM_10_, particulate matter 10 µm in diameter; NO_2_, nitrogen dioxide; SO_2_, sulfur dioxide. For categorical variables, number (n) and percentage (%) are presented. For continuous variables, mean (SD) is presented. ^a^ Others: ambulance emergency calls of poison, mental, digestion, and endocrine. ^b^ Time of day: daytime is from 08:00 to 19:00 for one day; nighttime is from 20:00 to 07:00 the next day.

**Table 2:** Associations of a mixture of air pollutants evaluated using quantile g-computation with all-cause and cause-specific ambulance emergency calls.

Ambulance Emergency Calls	Effect of Mixtures ψ (log RR, %)	ERR (%)	95% CI (%)	*p*-Value
**All-cause**	1.65	1.67	(0.12, 3.22)	0.03
Vascular disease	1.79	1.81	(0.25, 3.39)	0.02
Respiratory disease	2.22	2.25	(−0.15, 4.70)	0.07
Reproductive illness	1.75	1.77	(0.44, 3.11)	0.008
Injury	2.10	2.12	(0.56, 3.71)	0.008

Abbreviations: RR, relative risk; ERR, excessive relative risk; 95% CI, 95% confidence interval. Associations of an interquartile range (IQR) increase in PM_2.5_, PM_10_, Ozone, NO_2_, and SO_2_ exposure with ambulance emergency calls in multipollutant models. Models were adjusted for hourly temperature, hourly relative humidity, holiday, day of week, and calendar year.

**Table 3:** Associations of a mixture of air pollutants evaluated using quantile g-computation with all-cause and cause-specific ambulance emergency calls stratified according to age, sex, season, and time of day.

Subgroups	All-Cause	Vascular Disease	Respiratory Disease	Reproductive Illness	Injury
	ERR, % (95% CI, %)	ERR, % (95% CI, %)	ERR, % (95% CI, %)	ERR, % (95% CI, %)	ERR, % (95% CI, %)
**Sex**					
Male	1.62 (0.36, 2.87)	1.74 (−0.23, 3.72)	2.13 (−0.51, 4.78)	-	1.96 (−1.42, 5.31)
Female	1.63 (0.16, 3.11)	1.75 (0.20, 3.31)	2.14 (1.31, 2.92)	1.79 (−0.84, 4.17)	2.02 (−0.81, 4.69)
**Age group, years**					
Less than 20	1.61 (0.79, 2.42)	-	2.14 (0.00, 4.14)	1.79 (−0.47, 4.05)	1.92 (−1.30, 5.15)
20 to 64	1.61 (0.0, 3.30)	1.77 (0.00, 3.51)	2.12 (−0.11, 4.38)	1.77 (−1.33, 4.91)	1.90 (−2.80, 6.78)
Equal or more than 65	1.89 (0.17, 3.62)	1.87 (0.00, 3.82)	2.23 (−0.74, 5.16)	-	1.99 (0.02, 3.61)
**Season**					
Warm	3.06 (1.44, 4.58)	3.16 (1.00, 5.33)	3.03 (0.75, 5.20)	2.99 (0.42, 5.55)	3.83 (0.72, 6.91)
Cool	0.91 (−1.36, 3.22)	1.29 (−0.81, 3.39)	1.57 (−0.50, 3.62)	0.92 (−1.85, 3.68)	1.38 (−3.70, 6.35)
**Time of day**					
Daytime	1.84 (−0.31, 3.98)	2.00 (0.42, 3.56)	2.53 (0.81, 4.26)	2.21 (−0.81, 5.23)	1.92 (−1.62, 5.40)
Nighttime	1.41 (−0.27, 3.10)	1.48 (−1.03, 3.99)	1.91 (−1.64, 5.47)	1.56 (−1.52, 4.65)	2.55 (−1.70, 6.72)

Abbreviations: ERR, excessive relative risk; 95% CI, 95% confidence interval. Associations of an interquartile range (IQR) increase in PM_2.5_, PM_10_, Ozone, NO_2_, and SO_2_ exposure with ambulance emergency calls in multipollutant models. Models were adjusted for hourly temperature, hourly relative humidity, holiday, day of week, and calendar year.

## Data Availability

The datasets generated and/or analyzed during the current study are not publicly available owing to security protocols and privacy regulations, but they may be made available on reasonable request to the corresponding author.

## Supplementary Materials

The following supporting information can be downloaded at: https://www.mdpi.com/article/10.3390/toxics11110895/s1, Figure S1. Distribution of environmental monitors in Shenzhen city, China. Figure S2. Spearman correlations among air pollutants and meteorological factors. Figure S3. Cumulative concentration–response curves for the association of all-cause ambulance emergency calls with PM2.5, PM10, Ozone, NO_2_, and SO_2_ over lags 0–48 h in Shenzhen from 2013 to 2019. Figure S4. Lag structures for the associations of ambulance emergency calls with each interquartile range increase concentration in PM2.5, PM10, Ozone, NO_2_, and SO_2_ over lags 0–48 h in Shenzhen from 2013 to 2019. Figure S5. Weights represent the proportion of the positive or negative partial effect of each pollutant in the quantile g-computation model. Figure S6. Sensitivity analysis: exposure–response relationship between air pollutant mixtures and all-cause ambulance emergency calls in Shenzhen from 2013 to 2019. Figure S7. Sensitivity analysis: exposure–response relationship between air pollutant mixtures and cause-specific ambulance emergency calls in Shenzhen from 2013 to 2019. Figure S8. Sensitivity analysis: exposure–response relationship between air pollutant mixtures and sub-vascular ambulance emergency calls in Shenzhen from 2013 to 2019. Figure S9. Sensitivity analysis: exposure–response relationship between air pollutant mixtures and sub-respiratory ambulance emergency calls in Shenzhen from 2013 to 2019.
