# Supplementary material for: The Combined Effects of Hourly Multi-Pollutant on the Risk of Ambulance Emergency Calls: A Seven-Year Time Series Study
Source: Toxics. 2023 Oct 31;11(11):895. doi: 10.3390/toxics11110895 (PMC10675017; doi:10.3390/toxics11110895)
Supplement: Supplementary file 1 [file toxics-11-00895-s001.zip › toxics-2685706-supplementary.pdf]

toxics

MDPI

Supplementary Materials: The Combined Effects of Hourly Multi-Pollutant on the Risk of Ambulance Emergency Calls: A Seven-Year Time Series Study

Hanxu Shi, Qiang Zhou, Hongjuan Zhang, Shengzhi Sun, Junfeng Zhao, Yasha Wang, Jie Huang, Yinzi Jin, Zhijie Zheng, Rengyu Wu and Zhenyu Zhang

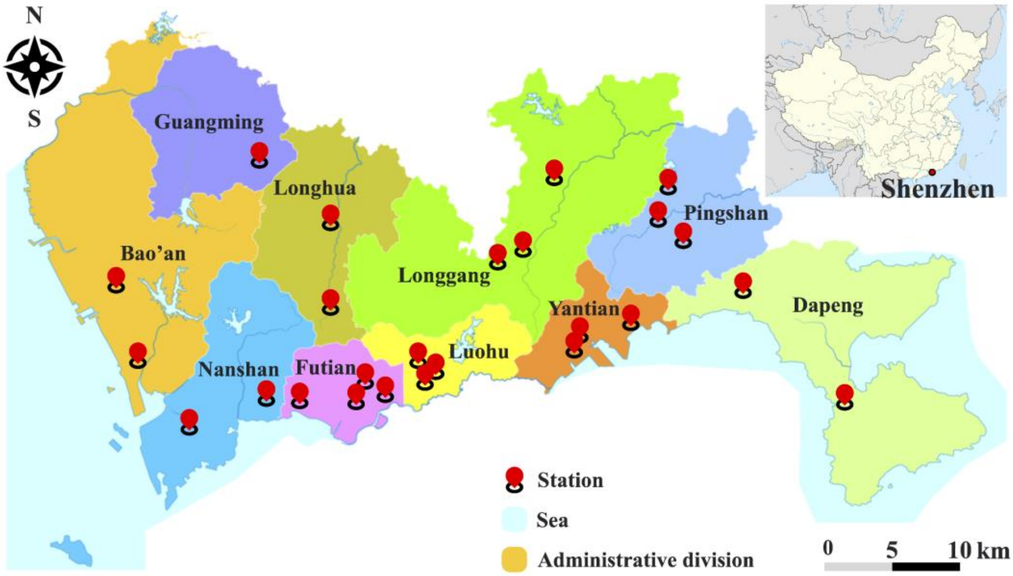

Figure S1. Distribution of environmental monitors in Shenzhen city, China.

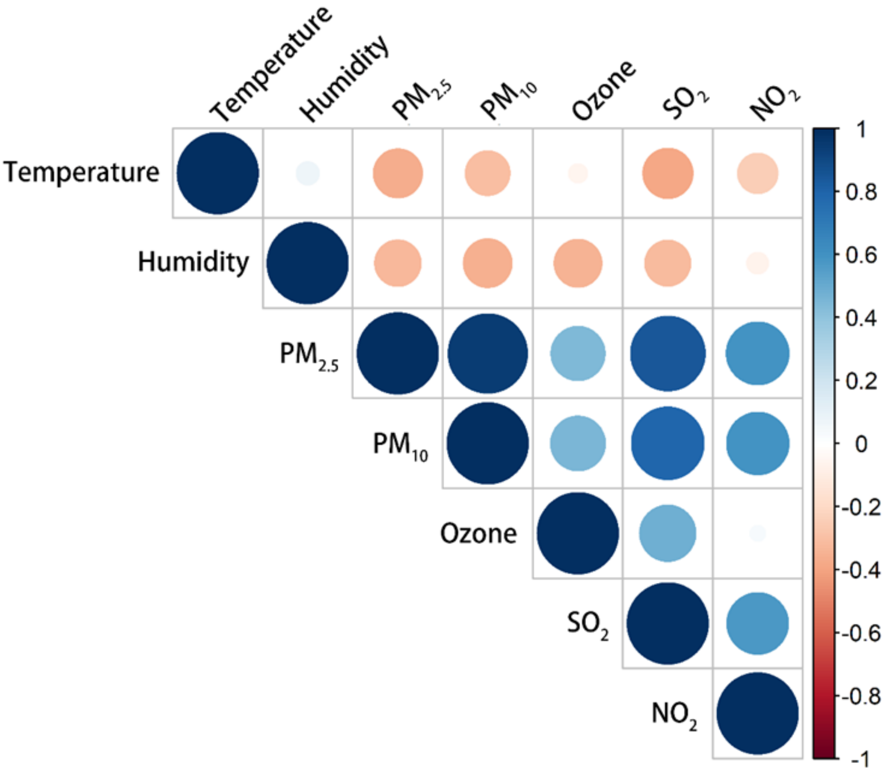

Figure S2. Spearman correlations among air pollutants and meteorological factors. Abbreviations: $PM_{2.5}$, PM_{10}, particulate matter less than 2.5,10 μm in diameter; NO_2, nitrogen dioxide; SO_2, sulfur dioxide.

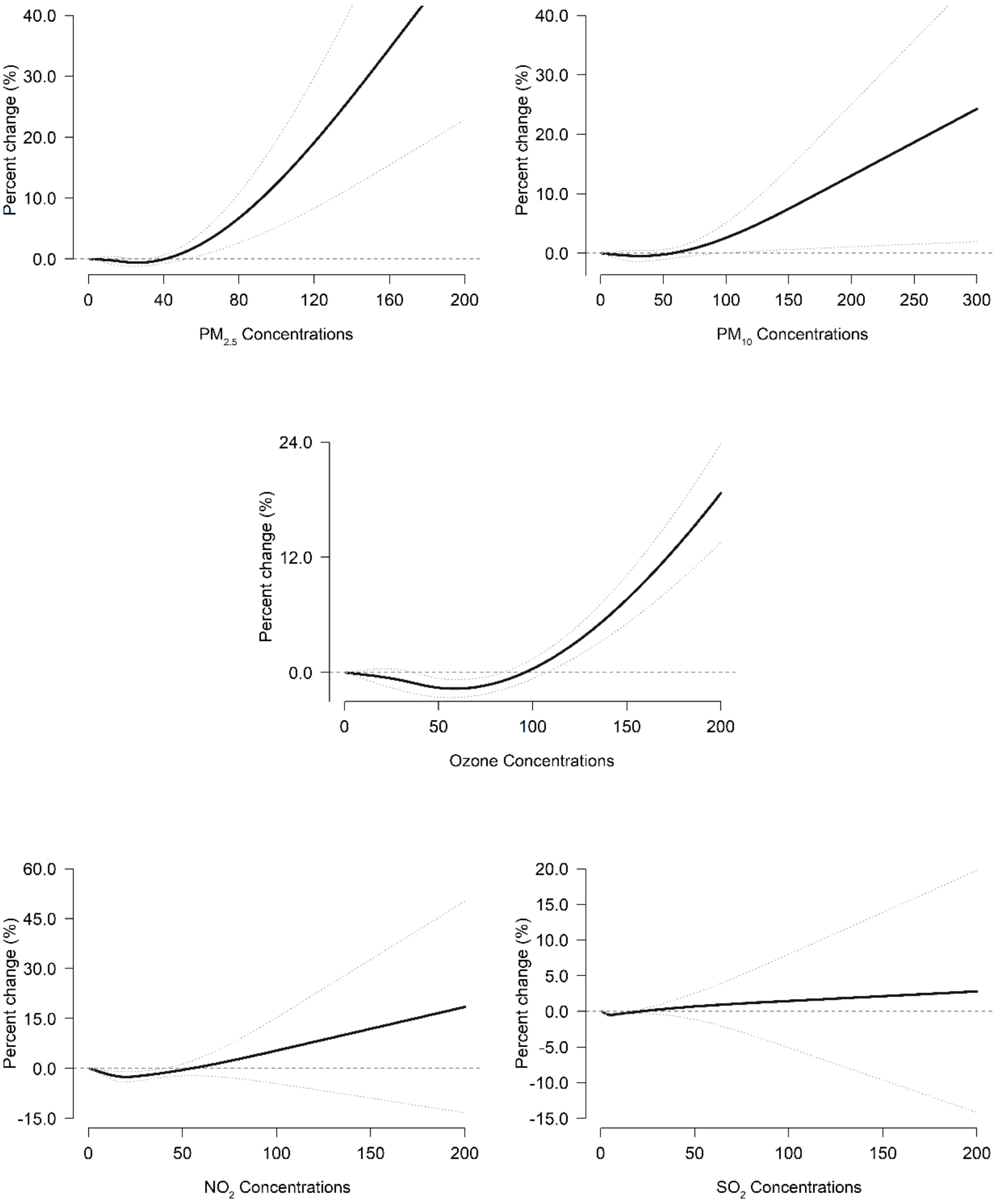

Figure S3. Cumulative concentration-response curves for the association of all-cause ambulance emergency calls with $PM_{2.5}$, PM_{10}, Ozone, NO_2, and SO_2 over lags 0-48h in Shenzhen from 2013-19. The cumulative exposure-response curves are calculated using a natural B-spline with two knots to model the exposure-response association. The black solid lines are the average percentage change in the risk of all-cause ambulance emergency calls, and the dotted lines are the 95% confidence intervals. Abbreviations: $PM_{2.5}$, PM_{10}, particulate matter less than 2.5,10 μm in diameter; NO_2, nitrogen dioxide; SO_2, sulfur dioxide.

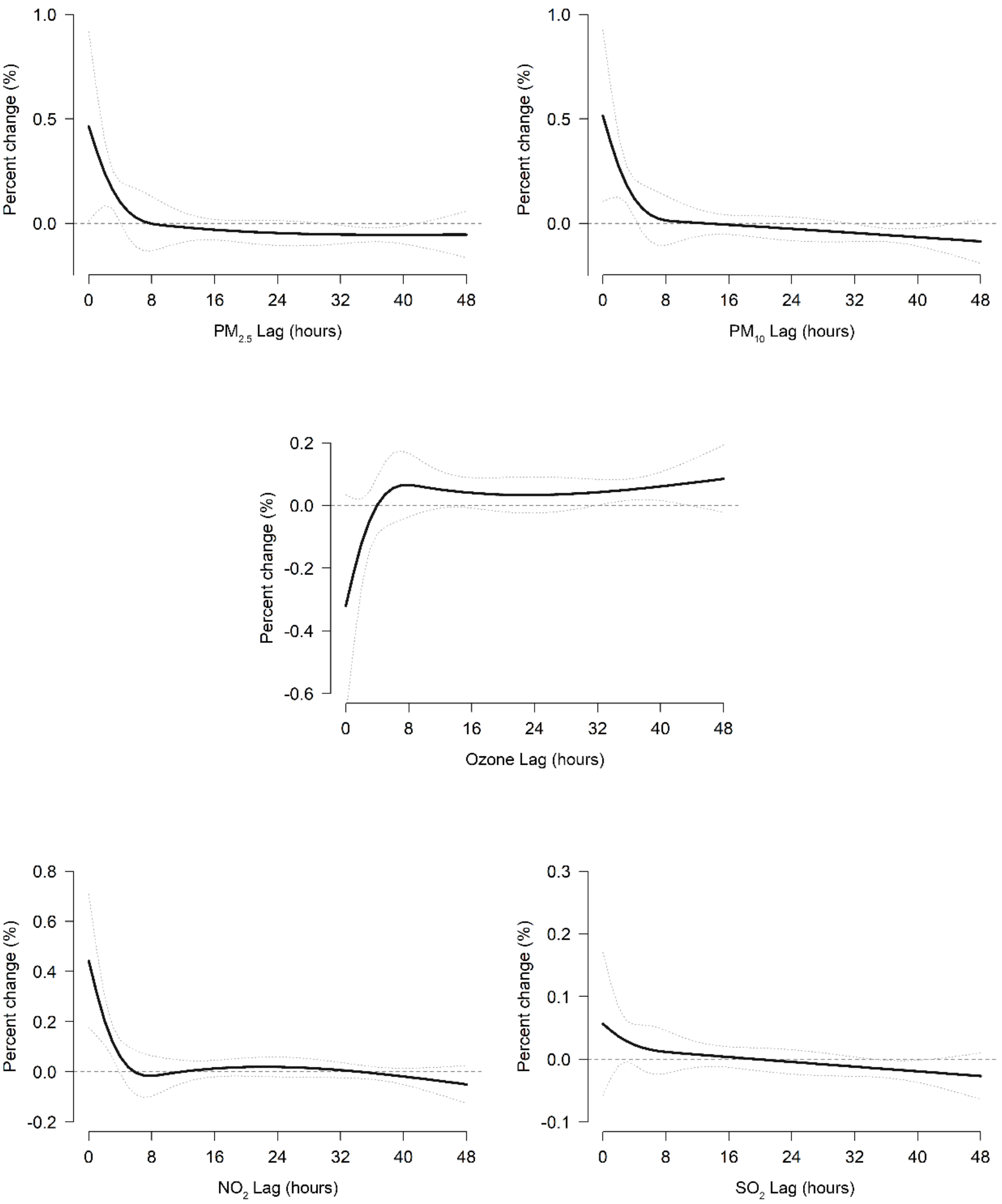

Figure S4. Lag structures for the associations of ambulance emergency calls with each interquartile range increase concentration in $PM_{2.5}$, PM_{10}, Ozone, NO_2, and SO_2 over lags 0-48h in Shenzhen from 2013-19. The overall lag structure curves are calculated using a linear with two knots placed on the log scale of lags to model the lag-response association. The solid black lines are the average percentage change in the risk of all-cause ambulance emergency calls with each IQR increase in $PM_{2.5}$, PM_{10}, Ozone, NO_2, and SO_2 and the dotted lines are the 95% confidence intervals. Abbreviations: IQR, interquartile range; $PM_{2.5}$, PM_{10}, particulate matter less than 2.5,10 μm in diameter; NO_2, nitrogen dioxide; SO_2, sulfur dioxide.

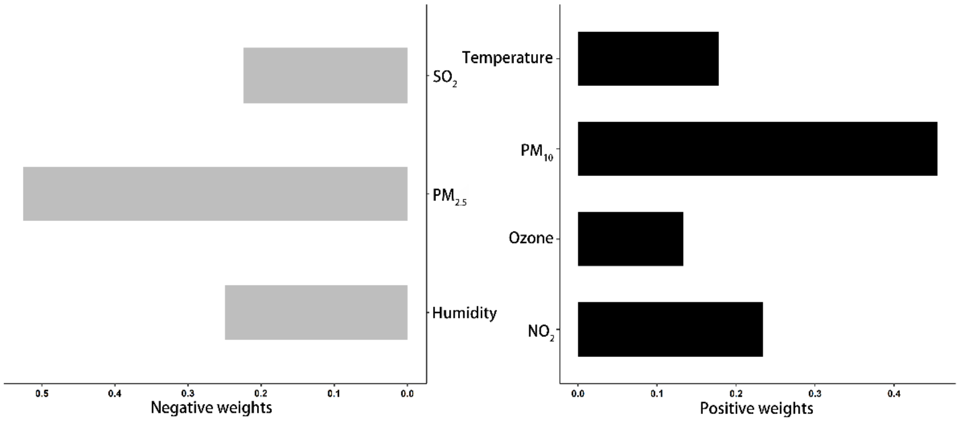

Figure S5. Weights representing the proportion of the positive or negative partial effect of each pollutant in quantile g-computation model. Abbreviations: $PM_{2.5}$, PM_{10}, particulate matter less than 2.5,10 μm in diameter; NO_2, nitrogen dioxide; SO_2, sulfur dioxide.

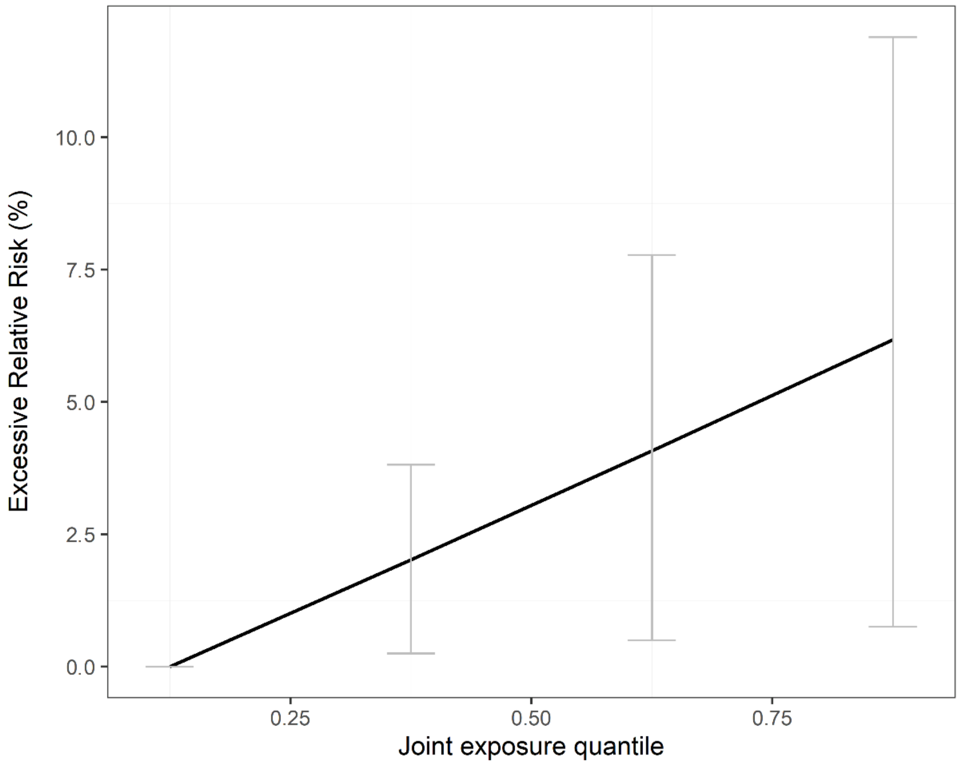

Figure S6. Sensitivity analysis: Exposure-response relationship between air pollutant mixtures and all-cause ambulance emergency calls in Shenzhen from 2013-19. The line is the exposure response curve, and the gray error bar is the confidence interval at each percentile. P-value <0.001 for linear relationship.

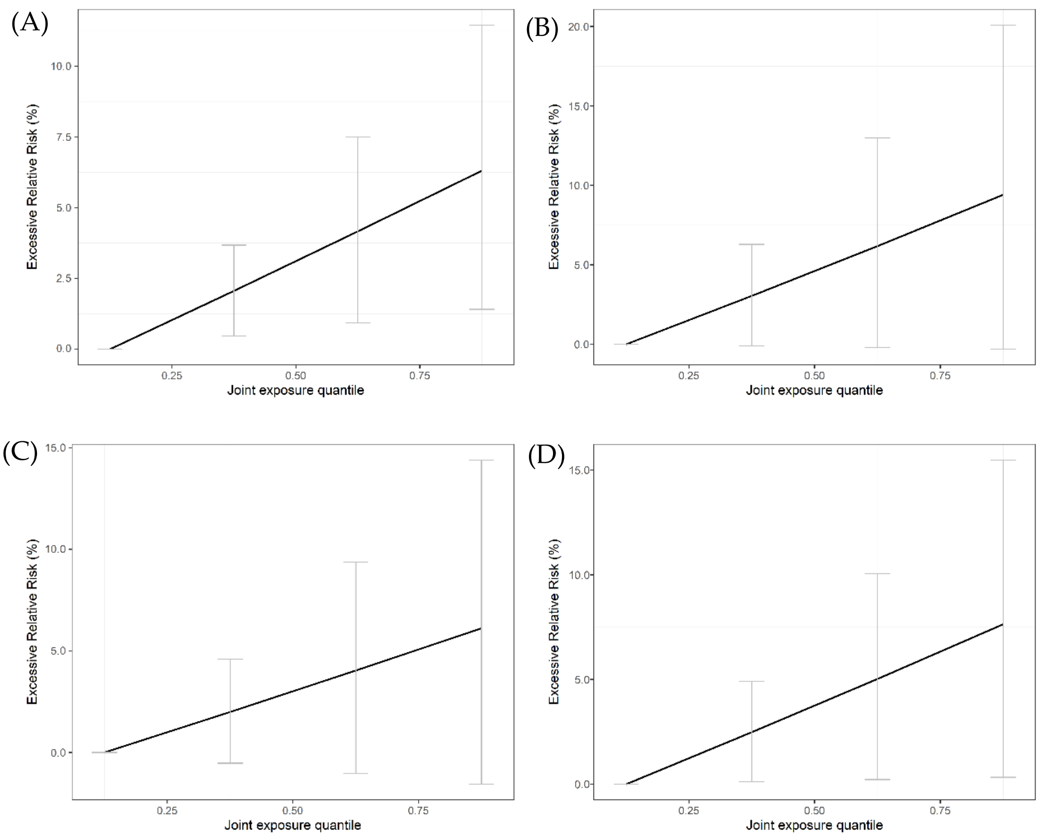

Figure S7. Sensitivity analysis: Exposure-response relationship between air pollutant mixtures and cause-specific ambulance emergency calls in Shenzhen from 2013-19. (A) represents vascular disease; (B) represents respiratory disease; (C) represents reproduction disease; (D) represents injury. The line is the exposure response curve, and the gray error bar is the confidence interval at each percentile. P-value < 0.001 for linear relationship.

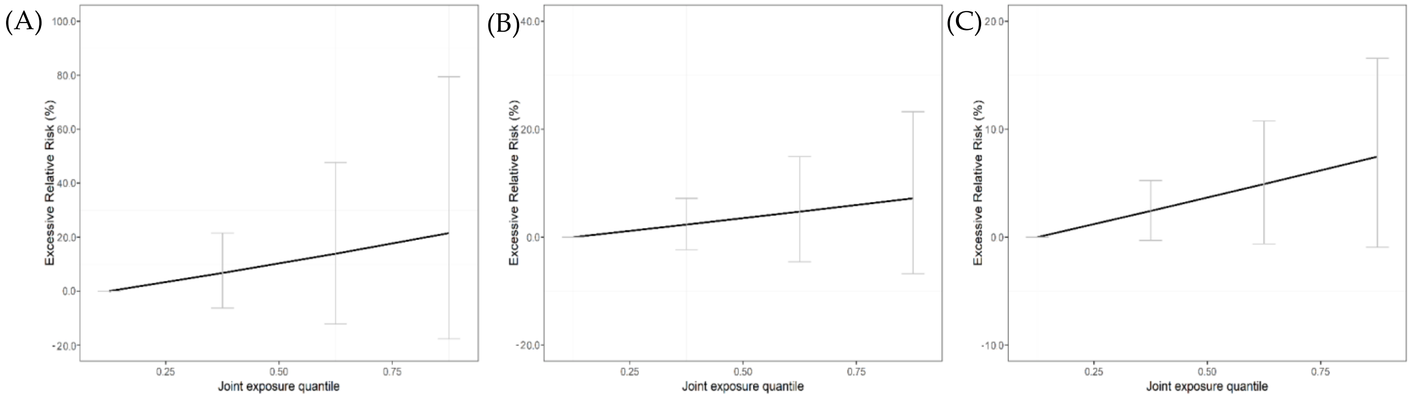

Figure S8. Sensitivity analysis: Exposure-response relationship between air pollutant mixtures and sub-vascular ambulance emergency calls in Shenzhen from 2013-19. (A) represents myocardial infarction (n=2,770); (B) represents acute coronary syndrome (n=30,892); (C) represents stroke (n=92,302). The line is the exposure response curve, and the gray error bar is the confidence interval at each percentile. P-value < 0.001 for linear relationship.

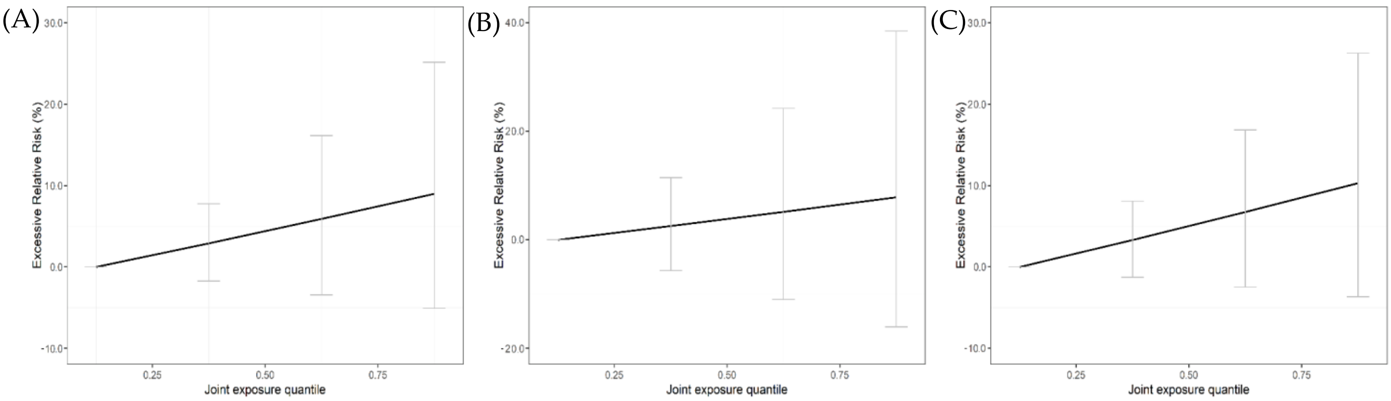

Figure S9. Sensitivity analysis: Exposure-response relationship between air pollutant mixtures and sub-respiratory ambulance emergency calls in Shenzhen from 2013-19. (A) represents pneumonia (n=31,159); (B) represents asthma (n=13,685); (C) represents upper respiratory infection (n=33,305). The line is the exposure response curve, and the gray error bar is the confidence interval at each percentile. P-value < 0.001 for linear relationship.
